# Cost-effectiveness of increased influenza vaccination uptake against readmissions of major adverse cardiac events in the US

**DOI:** 10.1371/journal.pone.0213499

**Published:** 2019-04-29

**Authors:** Samuel K. Peasah, Martin I. Meltzer, Michelle Vu, Danielle L. Moulia, Carolyn B. Bridges

**Affiliations:** 1 Department of Pharmacy Practice, College of Pharmacy, Mercer University, Atlanta, GA, United States of America; 2 Influenza Division, National Center for Immunization and Respiration Diseases, (CDC), Atlanta, GA, United States of America; 3 National Center for Emerging Zoonotic and Infectious Diseases, Centers for Disease Control and Prevention (CDC), Atlanta, GA, United States of America; 4 IHRC, Inc., Atlanta, GA, United States of America; 5 Immunization Services Division, National Center for Immunization and Respiratory Diseases, (CDC), Atlanta, GA, United States of America; Azienda Ospedaliero Universitaria Careggi, ITALY

## Abstract

**Background:**

Although influenza vaccination has been shown to reduce the incidence of major adverse cardiac events (MACE) among those with existing cardiovascular disease (CVD), in the 2015–16 season, coverage for persons with heart disease was only 48% in the US.

**Methods:**

We built a Monte Carlo (probabilistic) spreadsheet-based decision tree in 2018 to estimate the cost-effectiveness of increased influenza vaccination to prevent MACE readmissions. We based our model on current US influenza vaccination coverage of the estimated 493,750 US acute coronary syndrome (ACS) patients from the healthcare payer perspective. We excluded outpatient costs and time lost from work and included only hospitalization and vaccination costs. We also estimated the incremental cost/MACE case averted and incremental cost/QALY gained (ICER) if 75% hospitalized ACS patients were vaccinated by discharge and estimated the impact of increasing vaccination coverage incrementally by 5% up to 95% in a sensitivity analysis, among hospitalized adults aged ≥ 65 years and 18–64 years, and varying vaccine effectiveness from 30–40%.

**Result:**

At 75% vaccination coverage by discharge, vaccination was cost-saving from the healthcare payer perspective in adults ≥ 65 years and the ICER was $12,680/QALY (95% CI: 6,273–20,264) in adults 18–64 years and $2,400 (95% CI: -1,992–7,398) in all adults 18 + years. These resulted in ~ 500 (95% CI: 439–625) additional averted MACEs/year for all adult patients aged ≥18 years and added ~700 (95% CI: 578–825) QALYs. In the sensitivity analysis, vaccination becomes cost-saving in adults 18+years after about 80% vaccination rate. To achieve 75% vaccination rate in all adults aged ≥ 18 years will require an additional cost of $3 million. The effectiveness of the vaccine, cost of vaccination, and vaccination coverage rate had the most impact on the results.

**Conclusion:**

Increasing vaccination rate among hospitalized ACS patients has a favorable cost-effectiveness profile and becomes cost-saving when at least 80% are vaccinated.

## Introduction

Annual influenza vaccination has long been recommended for adults with certain medical conditions including cardiovascular disease (CVD), diabetes, and chronic lung disease, because of the increased risk of influenza-related complications, including hospitalization and death [1,2]. Influenza illness results in substantial economic impact, including costs related to outpatient and inpatient medical care, medications, lost productivity, decreased quality-of-life, and loss of life [3]. The association between influenza infection and acute myocardial infarction (AMI) or other major cardiac events has been established in the literature and CVD is the most commonly identified chronic medical condition among adults hospitalized with influenza [4–7]. A recent study found a significant association between influenza infection and acute myocardial infarction [8].

While multiple epidemiologic studies since the 1918 influenza pandemic have suggested an increased risk of severe influenza-related illness among persons with CVD, only more recent studies and meta-analyses have documented the benefits of influenza vaccination in preventing acute CVD-related outcomes among persons with existing CVD, specifically acute coronary syndrome (ACS) [9–13]. These studies define ACS to include acute ST-segment elevation myocardial infarction (STEMI), non-ST-segment elevation myocardial infarction (NSTEMI), and unstable angina (UA).

**Fig 1:**
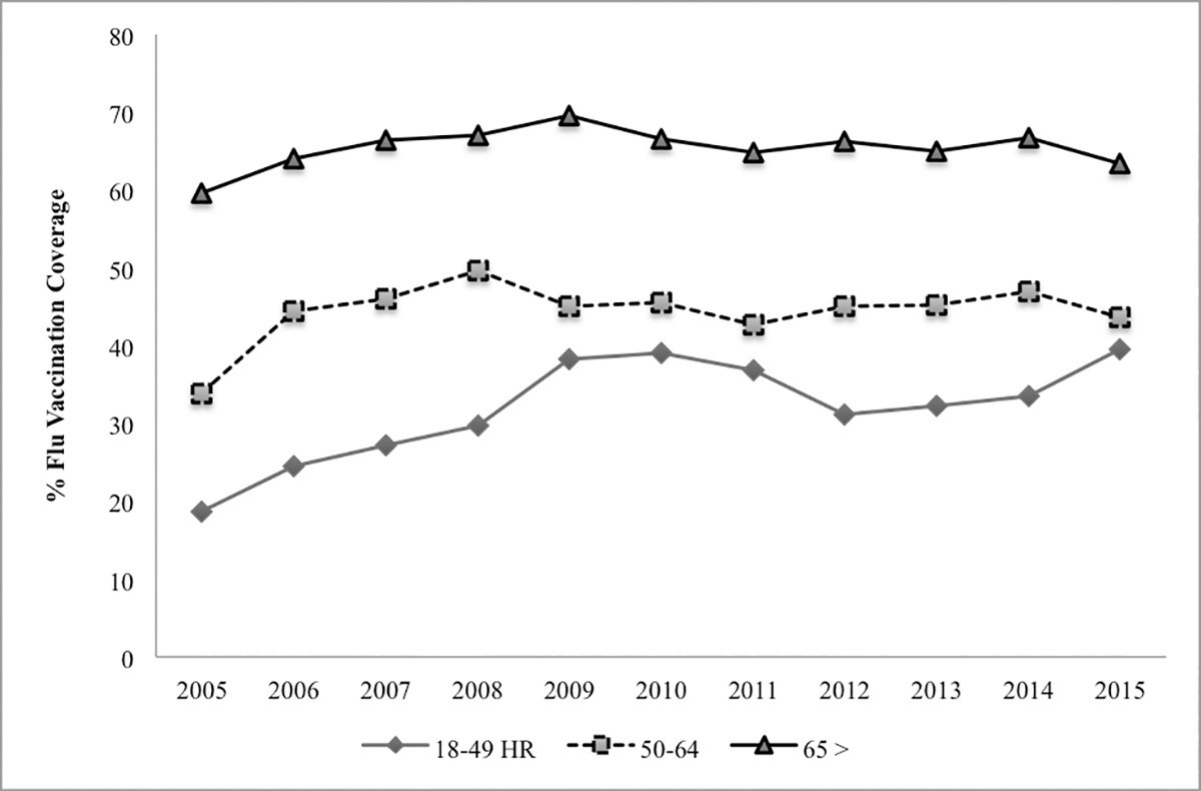
Influenza vaccination rate for high-risk (HR) patients from 2005–2015 [18].

Despite long standing recommendations for influenza vaccination of high risk patients by the Centers for Disease Control and Prevention’s (CDC) Advisory Committee on Immunization Practices (ACIP) [1], the American Heart Association, and the American College of Cardiology [14], only 50% of adults aged 18–64 years who have heart disease reported influenza vaccination in the 2012–2013 season [15,16], and only 48% of adults aged 18–64 years with any high risk condition for which influenza vaccination is recommended reported vaccination in 2015–16 (Fig 1). To evaluate these recommendations, we estimated the cost-effectiveness of increased influenza vaccination rate against readmitted major adverse cardiac events (MACE) of patients hospitalized for ACS at the current vaccination coverage rate (status quo) and assuming the World Health Organization (WHO) recommended 75% vaccination [17] for the elderly is attained by all adults hospitalized with ACS. Vaccine effectiveness (VE) of 30–40% against MACE was based on estimates from the meta-analysis by Udell et al. [13]. For the purpose of this study, MACE is defined, as in Ciszewski et al., as a composite measure of subsequent re-hospitalization for ACS due to myocardial infarction, coronary revascularization, or cardiovascular death [10].

## Methods

**Fig 2:**
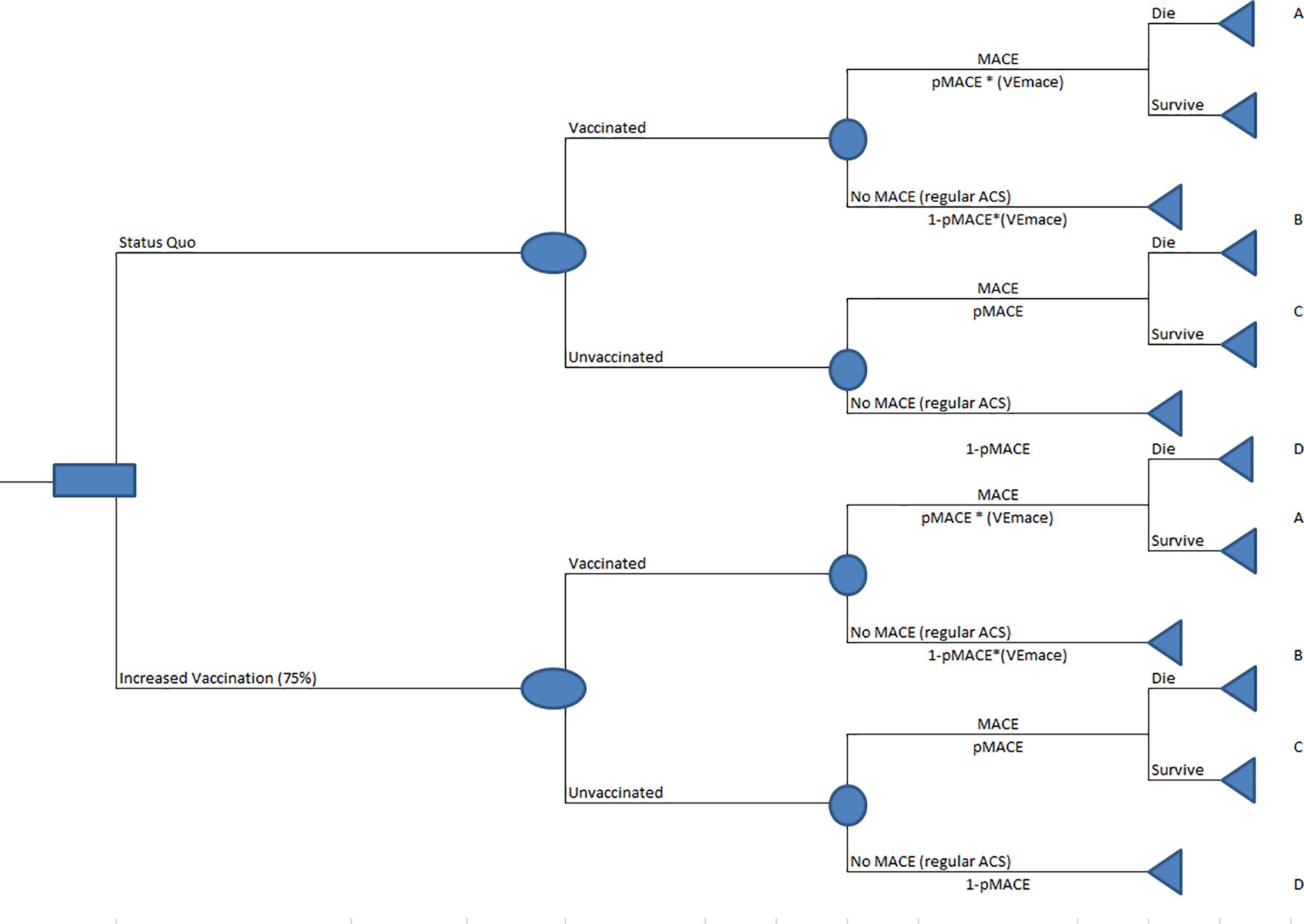
Decision tree model for cost effectiveness of influenza vaccination against MACE in ACS patients. pMACE = probability of Major Adverse Cardiac Event. No MACE (regular ACS) = All ACS patients without MACE readmission including readmissions not considered MACE. VEmace = Vaccine effectiveness against MACE. Increased Vaccination = vaccinating all patients who were not vaccinated at admission.

We developed a spreadsheet-based Monte Carlo probabilistic model (@Risk version 7 Palisade Corporations) [19] in 2018 to estimate the cost-effectiveness of increased influenza vaccination in a single year’s cohort (Fig 2) of ACS patients against readmitted MACE (S1 Table). The intended population is an estimated 625,000 ACS non-institutionalized (includes non-federal, short-term general, and other hospitals but excludes long-term care, rehabilitation and other institutions such as prisons) patient-discharges among persons, aged ≥18 years, in the United States [18,20,21]. Approximately 13% of these patients are classified as MACE after the ACS hospitalization [22]. Our model evaluates the protective benefit of influenza vaccination against MACE readmissions at the current vaccination rate among patients who report having cardiovascular disease and at 75% vaccination of admitted ACS patients. The health outcomes of interest are the number of MACE readmissions averted and the quality-adjusted life years (QALYs) gained. The model outputs are the incremental cost-effectiveness (ICER) of influenza vaccination against readmitted MACE at the current vaccination rate compared to a 75% vaccination rate pre-discharge in the natural units (cost/averted MACE) and in utilities (cost/QALY). Attaining 100% vaccination rate is ideal but unrealistic, vaccine effectiveness varies yearly, and increasing vaccination rates will increase cost of vaccination, therefore, we additionally conducted sensitivity analysis among these uncertainties for vaccination rates up to 95%.

### Statistical analysis

Incremental Cost Effective Ratio (ICER) = Incremental cost ((Sum of the total cost of MACE readmission and additional cost of vaccination in the increased vaccination scenario)–(Total cost of MACE readmissions in the status quo scenario))/Incremental effect (MACE averted (or QALYs gained) in the increased vaccination scenario-MACE averted (or QALYs gained) in the status quo scenario).

We used the healthcare payer perspective over a one-year period. This perspective estimates the cost of resources utilized by patients including clinician time, hospital bed, medications, and other auxiliary services but excludes costs related to outpatient care, productivity losses and loss of life. We chose this perspective because the payers are the most likely to incentivize implementation of efforts to expand vaccination of ACS patients. Additionally, we conducted both cost-effectiveness analysis and cost utility analysis (using QALYs) over a year from the payer’s perspective. We wanted to estimate the additional cost of reducing readmission rates of ACS due to influenza vaccination. We inflated all cost estimates to 2018 US dollars [23].

### Model inputs

#### Vaccine effectiveness

Three randomized control trials and a meta-analysis demonstrate the secondary protective effect of influenza vaccination against cardiovascular events in patients diagnosed with ACS [9–11, 13]. Gurfinkel, et al. assessed the impact of vaccination on a triple endpoint (cardiovascular death, myocardial infarction or severe recurrent ischemia) or on cardiovascular death alone over 6 months in one study and over 12 months in a follow-up study [11]. Phrommintikul, et al. also assessed the impact on MACEs, including deaths, hospitalization for ACS, stroke, and heart failure for 12 months [9]. Finally, Ciskewski, et al. assessed the impact of influenza vaccination on secondary prevention of coronary ischemic events (MACE and cardiovascular death separately) for 12 months [10]. Udell, et al. performed a meta-analysis of these trials and estimated a relative risk of 0.64 [95% CI 0.48–0.86] for MACE but found no statistically significant influenza vaccine effective against cardiovascular deaths as a separate endpoint [13]. Therefore, we used vaccine effectiveness against MACE of 36% (HR: 0.64 (95% CI: 0.48–0.86) [Table 1] and included vaccine effectiveness estimates of 30% and 40% in sensitivity analyses. We used the Beta probability distribution to describe the likelihood of any given level of vaccine effectiveness occurring. We based the model on trivalent influenza vaccination to reflect the vaccine used in these studies.

**Table 1:** Input variables for the model.

Input Variables	Literature Estimates	Variability	Distribution used	Source
**Total Number of Adult ACS**^**a**^ **discharges**	705,357		Fixed	AHA (21)
**Total Number of ACS in ≥65 years discharges**	438,889		Fixed	Healthcare utilization cost utilization project (HCUP) (20)
**Total number of ACS in 18 to 64 years discharges**	266,468		Fixed	HCUP (20)
**Readmission for ACS**	0.21		Fixed	Menzin (24)
**Vaccine Effectiveness against MACE**^**b**^ **(Hazard Ratio)**	36% (0.64)	0.48–0.86^e^	Beta	Udell (13)
**Vaccination Coverage in ≥65 years (for high risk patients)**	0.634	0.008^d^	Normal	CDC FluVAX (18)
**Vaccination Coverage in 18 to 64 years (for high risk patients)**	0.481	0.012^d^	Normal	O’Halloran (16)
**Probability of MACE Admission in 18 to 64 years**^**c**^	0.033		Fixed	Korsnes (26)
Probability of MACE Admission in ≥65 years*	0.033		Fixed	Korsnes (26)
**Probability of MACE deaths**	0.0862	0.0108–0.27^e^	Beta	Sribhutorn (30)
**Utilities**	0.76	0.584–0.974^e^	Normal	Lewis EF (28)
**Life expectancy at birth**	78.6		Fixed	CDC 2016 (29)
**Life expectancy at 75 years**	12.2		Fixed	CDC 2016 (29)
**Cost of MACE hospitalization (18+) i.e. everybody in the cohort**	$20,246	157^d^	Gamma	HCUP (20)
**Cost of MACE hospitalization (18–64)**	$21,156	262^d^	Gamma	HCUP (20)
**Cost of MACE hospitalization (65+)**	$19,543	181^d^	Gamma	HCUP (20)
**Vaccination cost**	$21.57	$6.63^d^	Normal	Singhal and Zhang(27)
**Proportion of MACE among hospitalized discharges by age group** **18–44 years** **45–64 years** **65–84 years** **85+ years**	0.0523 0.3823 0.4382 0.1270			HCUP (20)

a. ACS = Acute coronary syndrome.

b. MACE = Major adverse cardiac event

c. Probability of MACE hospitalization within one year of ACS discharge

d. SD = Standard deviation or standard error

e. CI = Confidence interval

#### Vaccine coverage

We used 63.4% vaccine coverage (SD 8%) for adults’ aged ≥ 65 years and 48% (SD 12%) for 18–64 years (Table 1). These estimates were based on 2015–16 influenza season CDC vaccination coverage surveillance [18] for older adults’ aged ≥ 65 years and for high-risk adults 18–64 years old. Influenza vaccination coverage for adults in the United States has been relatively stable over several years (Fig 1).

#### Number of ACS patients

Our model assumes that patients were vaccinated once and since hospital discharges include patients who are readmitted, we estimated the number of unique ACS hospitalized patients (493,750) by subtracting estimated readmissions (21%), [24] from the total hospital discharges (625,000). The American Heart Association (AHA) estimates 625,000 ACS [20,21] yearly discharges of adult patients ≥ 18 years based only on primary diagnosis of ACS [ICD-9 codes of 410 (AMI), 411 (other acute and subacute forms of ischemic heart disease) or ICD-10 codes of I20 (angina pectoris), I21(STEMI & NSTEMI), or I22 (subsequent STEMI & NSTEMI) [Table 1]. We estimated the proportion of adults hospitalized with ACS who are 18–64 years versus ≥65 years based on proportions in the HCUP discharge data. The current discharged estimates (493,750 unique individuals) exclude those who were not hospitalized because of prior influenza vaccination, we therefore adjusted the number of ACS admissions (i.e. 493,750*100/70 = 705,357) using CDC estimates of vaccine effectiveness against hospitalization (37% for adults’ ≥65years old and 30% for adults ≥18 years old) [25]. The final 705,357 used represents the number of unique individuals who would have been hospitalized if none had received the influenza vaccination.

#### Proportion of MACEs post ACS hospitalization

We used two estimates of MACE per year post-ACS hospitalization; 3.3% from MarketScan dataset by Korsnes, et al. [26] and 12.9% from Liu et al. of ACS patients without diabetes [22]. We used the conservative estimate of 3.3% in the model and 12.9% in sensitivity analyses (Table 1).

#### Cost data

The hospitalization cost of MACE was derived from HCUP [20] using hospitalization cost of AMI, which constitutes the majority of MACE cases including deaths; this estimated cost of nonfatal MACE hospitalizations from Korsenes, et al. [26]. HCUP provides cost data by age group (18–44 years, 45–64 years, 65–84 years, and ≥85years). The total cost for all ages, 18–64 years, or ≥ 65years was calculated by using the HCUP age group category cost and the proportion of discharges in each HCUP age group. The cost of hospitalization for all ages was estimated to be $20, 246 ($23,040 in 2018 US$). The calculated cost for patient’s aged ≥65 years was $19,543 ($22,241 in 2018 US$) and for 18–64 years, $21,157 ($24,077 in 2018 US$). The cost of vaccination was from Singhal and Zhang [27], which estimated the cost of vaccination from claims data using MarketScan dataset at three sites of care: physicians’ offices, pharmacies and other sites. Our model estimated the cost of vaccination based on the other sites estimate (Table 1). We used gamma distribution for cost of admission and normal distribution for cost of vaccination.

#### Utilities

Life years gained, from potential averted deaths due to averted MACEs, was adjusted using utilities (0.76±0.22) of US patients after myocardial infarction [28]. We used 2016 US life expectancy at birth for the 18–64 year group, and life expectancy at 75 (12.2 years) for the ≥65 year group. [29] We used 55 years to represent the average age of the 18–64 year group based on the proportions in the HCUP MACE discharges and 75 years for the ≥65 years group. The probability of death from MACE is estimated at 8.6% [30].

### Sensitivity analysis

We used probabilistic sensitivity analysis, taking into account the parameter uncertainties in the model jointly. We also performed one-way sensitivity analyses on all parameters by using ±20% which will account for the fixed variables in the model. Additional one-way sensitivity analysis included using 12.9% prevalence rate [15] for MACE instead of the conservative 3.3% used in the model, [26] and changing readmission rate of ACS from 21% to 6.8% (as estimated by Arnold et al.) [31] Finally, we conducted two two-way sensitivity analyses. One model described the impact of increasing vaccination cost per person (in increments of $5) on incremental cost effectiveness ratios (ICER) at different vaccine effectiveness rates (30%, 36% (current), and 40%). Another model described the impact of increasing average vaccination coverage in all persons aged ≥18 years (vaccination coverage in ≥65 and 18–64 years were increased by increments of 5% from 75% to 95%) on ICER at different vaccine effectiveness rates (30%, 36% (current), and 40%). In practice, in order to increase vaccination rates, vaccinating those currently unvaccinated may require more resources (i.e., marginal costs increase). This will increase the average cost.

## Results

In the base case analysis (current vaccination rate vs. 75% vaccination rate), an additional ~500 (95% CI: 578–825) MACEs were averted at vaccine effectiveness of 36% against MACE and vaccine coverage of 63.4% for persons aged ≥ 65 years and 48% for persons aged 18–64 year. Similarly, the number of QALYs added will be ~600 (95% CI: 578–825). The additional influenza vaccination cost was $3 million (Table 2).

**Table 2:** Probabilistic model outputs.

	**Mean** **(95% confidence interval)**		
	Ages 18+	Ages 65+	Ages 18–64
**Scenario: Status Quo (only some admitted ACS**^**a**^ **patients vaccinated)**			
**Total cost of MACE**^**b**^ **readmissions**	$108,349,700 (95,709,450–125,869,100)	$63,638,670 (54,498,330–78,303,220)	$44,711,060 (38,371,420–54,987,500)
**# of MACEs Averted**	**956** (821–1,098)	**657** (559–760)	**299** (249–352)
**# of Life Years Added**	**1,299** (1,095–1,525)	**691** (580–815)	**609** (500–727)
**# of QALYs Added**	**988** (832–1,159)	**525** (441–619)	**463** (380–553)
**Scenario: Increased vaccination (75%)**			
**Total cost of MACE readmissions + additional vaccination**	**$109,888,700** (96,724,380–127,438,000)	**$60,676,360** (51,925,140–74,377,250)	**$49,212,340** (41,968,480–60,365,470)
**Additional cost of vaccination**	**$3,146,118** (1,415,312–4,954,386)	**$1,421,377** (587,841–2,380,344)	**$1,724,740** (785,226–2,705,031)
**# of MACEs Averted**	**1,486** (1,293–1,679)	**821** (715–928)	**665** (579–751)
**# of Life Years Added**	**2,216** (1,894–2,560)	**864** (738–998)	**1,352** (1,156–1,562)
**# of QALYs Added**	**1,684** (1,439–1,946)	**656** (561–758)	**1,028** (878–1,187)
**Cost per additional MACE Averted** **(ICER)**^**c**^	**$3,220** ((2,585)-9,778)	**($17,985)** ((25,521)-(11,521))	**$12,680** (6,273–20,264)
**Cost per additional QALY added** **(ICER)**^**c**^	**$2,432** ((1,992)-7,398)	**($22,552)** ((32,266)-(14,316))	**$12,680** (6,273–20,264)

a. ACS = Acute coronary syndrome

b. MACE = Major Adverse Cardiac Event

c. ICER = Incremental Cost Effectiveness Ratio which is the additional cost for an additional MACE case averted when comparing vaccinating all ACS patients to the status quo

The ICER per MACE averted was ~$3,000 (95%CI: -2,585–9,778) and ICER per QALY was ~$2,400(95%CI: -1992-7,398). The additional vaccination was cost-saving in adults aged ≥ 65 years but $~12,700/QALY in adults aged 18–64 years. (Table 2).

**Fig 3:**
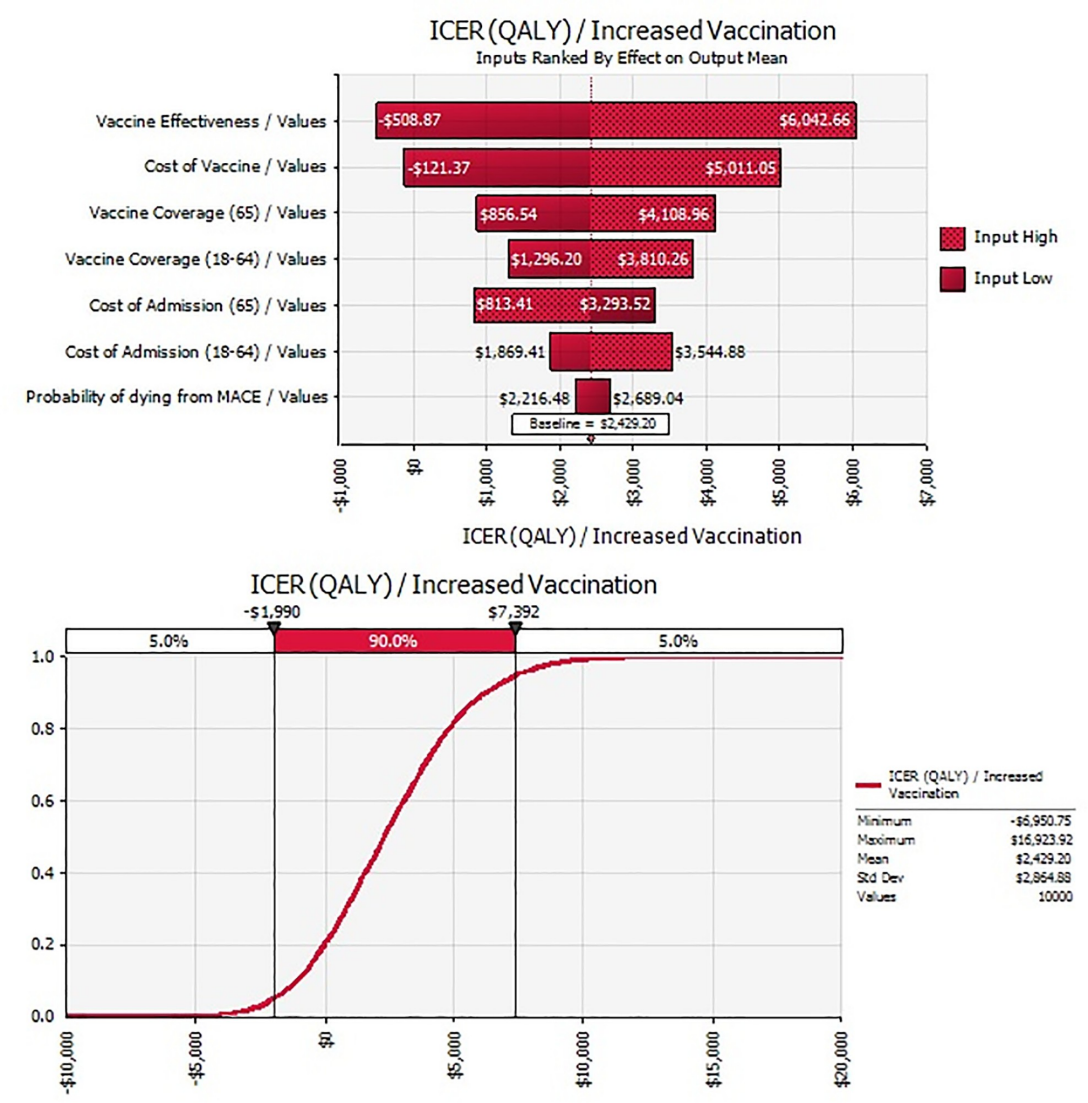
Tornado diagram of parameter sensitivity to ICER/QALY.

In the one-way sensitivity analysis of all parameters in the model and as illustrated in the tornado diagram (Fig 3), the variables with the most impact are vaccine effectiveness, cost of vaccine, vaccine coverage rate, and the cost of admission.

**Fig 4:**
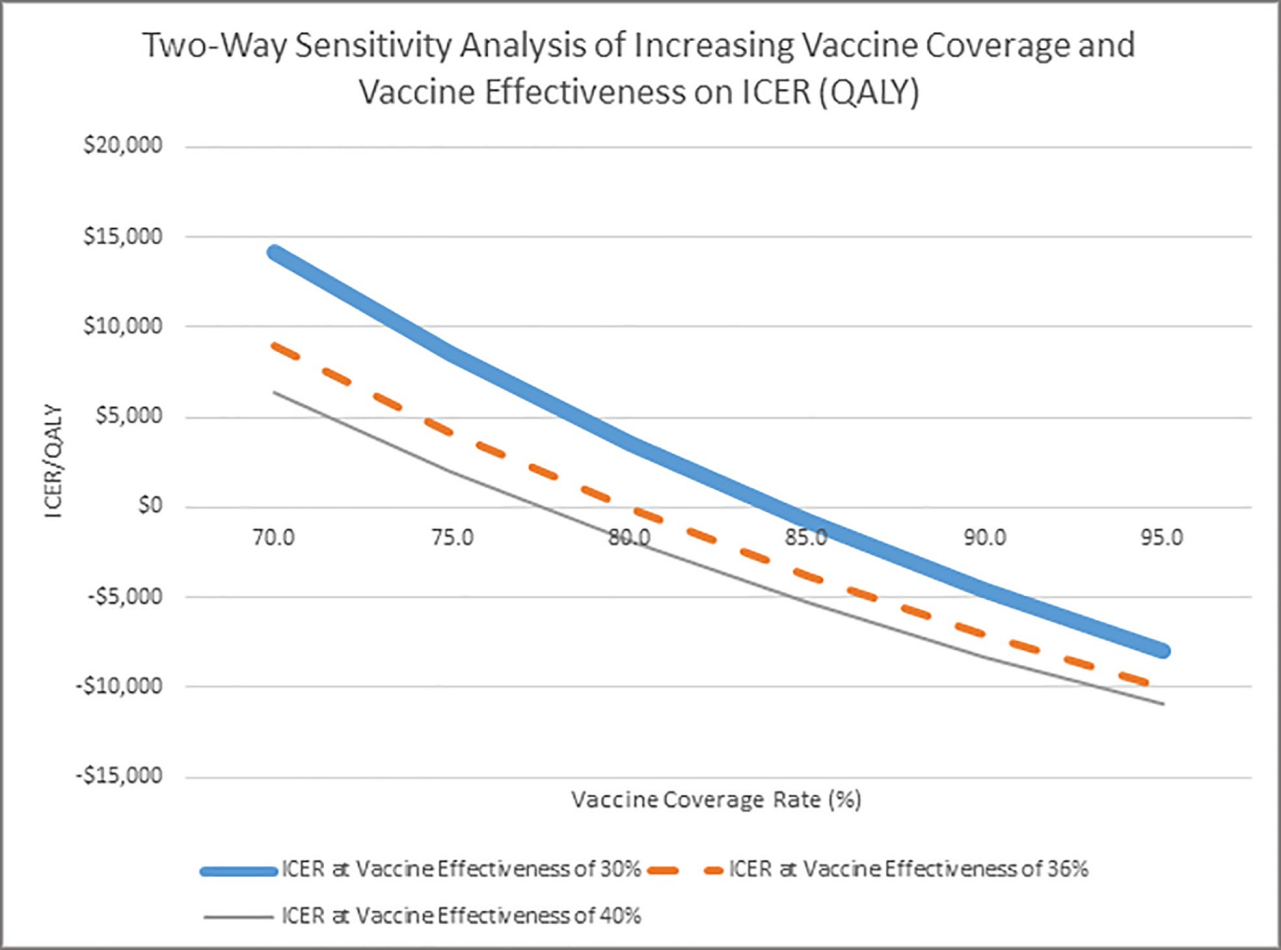
Impact of vaccine effectiveness and cost of vaccination on incremental cost effectiveness ratios (ICER). The cost of vaccination per person (from $20 and increasing by $5). ICER is the incremental cost effectiveness ratio of all 18+ adults vaccinated using status quo as reference.

**Fig 5:**
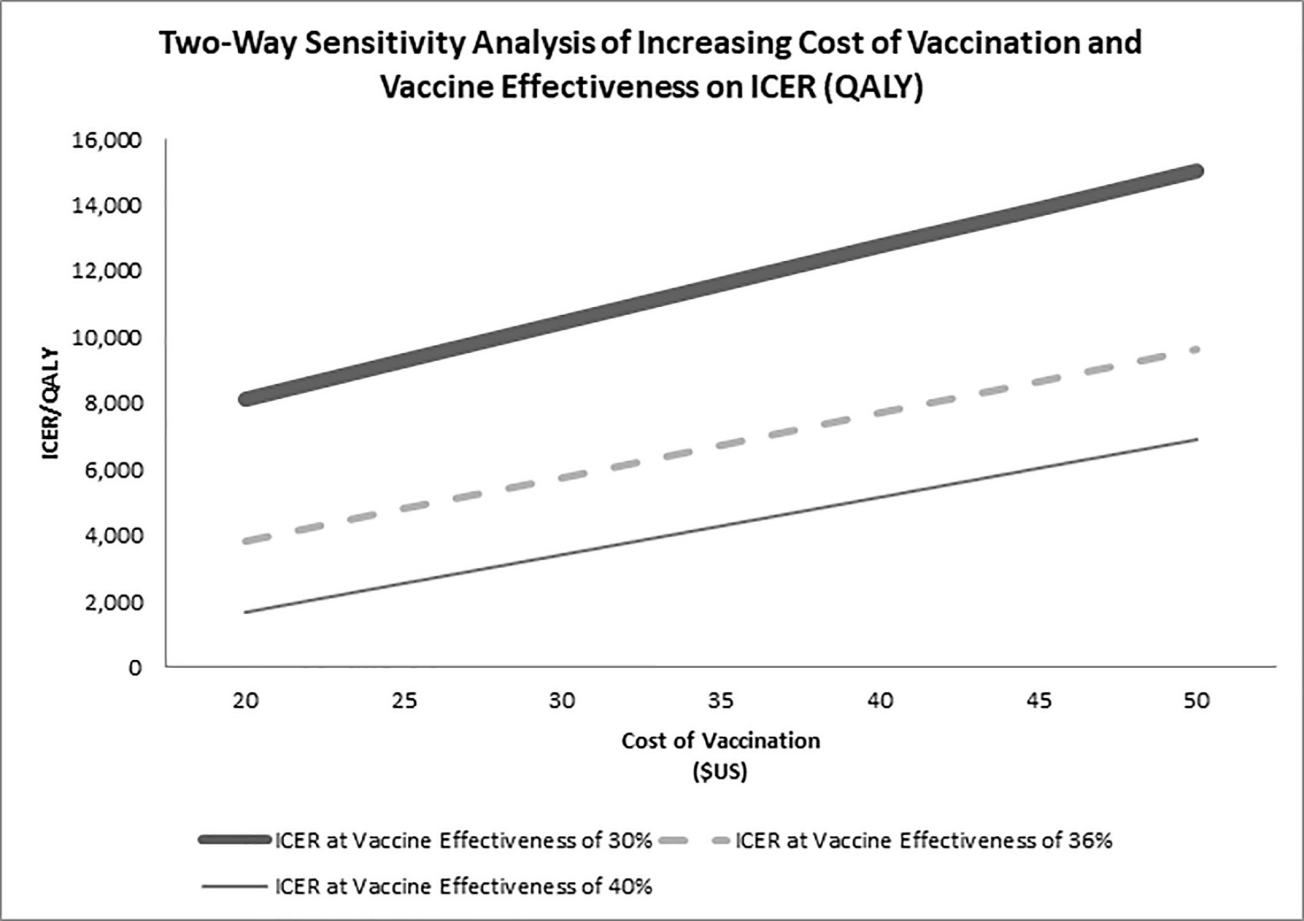
Impact of vaccine effectiveness and vaccination coverage (all persons 18+) on incremental cost effectiveness ratios (ICER).

Additionally, changing the probability of MACE from 3.3 to 12.9% did not have much impact on the results. In the two-way sensitivity analysis of changes in vaccination cost and vaccine effectiveness (Fig 4), vaccination remained less than $16,000/QALY even if the cost of vaccination was doubled to $50 and vaccination effectiveness is estimated at 30%. In the second two-way sensitivity analysis, vaccination is cost-saving after ~78% are vaccinated (at vaccine effectiveness of 40%), ~80% for vaccine effectiveness of 36%, and ~85% at vaccine effectiveness of 30%. (Fig 5).

## Discussion

We estimated increasing vaccination from the current rate to 75% of ACS hospitalized patients against MACE to be cost-saving for adults aged ≥ 65 years and favorable economically for adults aged 18–64 years. In a sensitivity analysis, the impact of increasing vaccination rate up to 95% was cost-saving for all adults after 80%.

The cost-effectiveness of influenza vaccination against additional use of healthcare resources has been established in the literature. For example, Patterson et al. reported, from the healthcare perspective, a cost-effectiveness ratio of $34,610 per life-year saved for adults ≥50 years, and $13,084 per life-year saved for adults ≥ 65 years who get vaccinated against influenza in the emergency department [32]. In terms of benefit-cost-ratio (BCR), You et al. reported BCR of 6.39 if influenza vaccination is compared with no vaccination and BCR of 5.10 if influenza vaccination is combined with pneumonia vaccination against no vaccination in elderly people [33]. Mullooly, et al. and others found vaccination to be cost-saving in adults [34, 35]. Additionally, Prosser, et al. [36] calculated the cost-effectiveness of vaccination against the 2009 pandemic influenza A (H1N1) as cost-saving in persons aged 6 months to 64 years at influenza attack rates ≥15%, but can range from $8,000-$52,000/QALY depending on vaccination setting and risk-status.

Regarding the clinical benefits of influenza vaccination against MACE, the exact biological mechanism is unknown. However, studies have posited that inflammation from acute respiratory distress (influenza virus A and B) may trigger atherosclerotic plaque rupture resulting in AMI [11, 12]. Multifactorial mediators contribute to MACE among persons with existing CVD. Triggers of MACE include sympathetic stimulation, endothelial dysfunction, and procoagulant activation [12]. By preventing cardiovascular stress and inflammation due to acute influenza illness with fever, increased work of breathing, and inflammation, influenza vaccination may provide cardioprotective benefits and reduce the risk of adverse cardiovascular events.

Heart disease has been estimated to increase the risk of influenza-related hospitalization 2.7 fold [2]. The clinical benefits and economic value from our model findings suggest that initiatives to increase vaccination rates among patients with CVD could be good investments by third party payers and policy makers, especially for ACS-hospitalized patients.

For most adults aged ≥ 65 years, the cost of influenza vaccination should not be a significant barrier. Medicare Part B includes influenza vaccination at no copay to beneficiaries and all but one state (Florida) include influenza vaccination as a benefit for patients on Medicaid [37]. The cost of influenza vaccination is commonly reported as a barrier to vaccination in surveys of patients. Use of standing orders for vaccination has been shown to significantly improve vaccination rates and may be considered as a strategy for vaccination of patients with CVD prior to hospital discharge and when seen for clinical outpatient care [38]. Pharmacists can also play a role by reminding patients on cardiovascular medications of the need for annual influenza vaccination.

This study is subject to the following limitations: our model was based on estimates from a meta-analysis of clinical trials that did not include cost data. Thus, costs were estimated using published data from the literature. Furthermore, many costs for patients, including costs for outpatient follow-up, and treatment after MACE hospitalization, nursing home, or other post-hospitalization care after a MACE hospitalization were not included in the analyses, thus our estimate is a conservative estimate of the cost-effectiveness of influenza vaccination of patients with CVD. Our one-year horizon also implies that we are ignoring addition cost of survival from MACE in subsequent years. Additionally, we recognize that patients vaccinated at discharge might not have the full protection against influenza-related readmissions within may be the first two weeks. In the sensitivity analysis, we accounted for that by using lower vaccine effectiveness.

## Conclusion

Adding influenza vaccination prior to discharge for patients admitted for ACS could substantially reduce subsequent re-hospitalization due to MACE. Achieving a vaccination rate of at least 80% could be cost-saving. All medical providers of patients with cardiovascular disease, including pharmacists, have a role in assessing patients’ vaccination status at each clinical encounter, including hospital discharge, providing a clear recommendation for influenza vaccination, and offering influenza vaccination and other vaccines as indicated. Medical primary care providers, [39] specialty providers and other vaccine providers, including pharmacists could prevent MACEs during the influenza season by stocking influenza vaccines and recommending influenza vaccination for all adult patients with CVD each year.

## Supporting information

S1 Table Microsoft excel sheets containing model. (XLSX)
